# Statistical analysis of arthroplasty data: II. Guidelines

**DOI:** 10.3109/17453674.2011.588863

**Published:** 2011-07-08

**Authors:** Jonas Ranstam, Johan Kärrholm, Pekka Pulkkinen, Keijo Mäkelä, Birgitte Espehaug, Alma Becic Pedersen, Frank Mehnert, Ove Furnes

**Affiliations:** ^1^Swedish National Competence Center Musculoskeletal Disorders, Skåne University Hospital, Lund, The Swedish Knee Arthroplasty Register, and Lund University; ^2^The Swedish Hip Arthroplasty Register, Sahlgrenska University Hospital and Göteborg University, Göteborg, Sweden; ^3^The Finnish Arthroplasty Register and Department of Public Health, University of Helsinki; ^4^The Finnish Arthroplasty Register and Turku University Central Hospital, Turku, Finland; ^5^The Norwegian Arthroplasty Register, Department of Orthopaedic Surgery, Haukeland University Hospital, Bergen, Norway; ^6^The Danish Hip and Knee Arthroplasty Register, Department of Clinical Epidemiology, Competence Center North, Aarhus University Hospital, Aarhus, Denmark; ^7^Department of Surgical Sciences, University of Bergen, Norway

## Abstract

It is envisaged that guidelines for statistical analysis and presentation of results will improve the quality and value of research. The Nordic Arthroplasty Register Association (NARA) has therefore developed guidelines for the statistical analysis of arthroplasty register data. The guidelines are divided into two parts, one with an introduction and a discussion of the background to the guidelines (Ranstam et al. 2011a, see pages x-y in this issue), and this one with a more technical statistical discussion on how specific problems can be handled. This second part contains (1) recommendations for the interpretation of methods used to calculate survival, (2) recommendations on howto deal with bilateral observations, and (3) a discussion of problems and pitfalls associated with analysis of factors that influence survival or comparisons between outcomes extracted from different hospitals.

This paper on statistical guidelines for analysis of arthroplasty register data is divided into 4 sections, each one addressing methodological issues. The sections include recommendations about (1) competing risk problems, (2) detecting and handling departures from the proportional hazards assumption, (3) bilateral observation, and (4) revision rate ranking.

An introduction to and background description of these problem areas is presented in Part I. This second part contains a more technical discussion.

## 1. Competing risk

The endpoint analyzed in arthroplasty registries often consists of two distinct events: revision and death. The latter, of course, always precludes the occurrence of a subsequent revision. It can be argued that the presence of a risk of a competing event (competing risk) may bias Kaplan-Meier (KM) survival estimates (Biau et al. 2007). The reason for this is that the validity of the KM method rests on the assumption of identical revision risk in censored and uncensored patients. If censored patients cannot be revised, which is the case with patients who are censored because of death, revision risk will be overestimated.

To obtain a more valid estimate of the revision risk, the cumulative incidence function can be used (Gooley et al. 1999). Here, patients' deaths can be considered to be competing events, while patients who are alive and unrevised at the end of follow-up can be censored. The method is described using an example.

### Example 1. Five patients with primary total hip arthroplasty

Consider a simple study of 5 patients with primary total hip arthroplasty who are followed for 10 years (Table 1, column 3), and assume that death is considered a competing event. The events studied are thus implant failure and death.

**Table 1.** Illustration of data censoring and estimation of implant failure using the Kaplan-Meier (KM) and cumulative incidence methods

			KM method			Cumulative incidence method				
A	B	C	D	E	F	G	H	I	J	K
2	3	Dead	Censored	0 × 1/5	0	Competing event	0 × 1/5	0	1 × 1/5	20
3	5	Revised	Event	1 × 1/4	25	Event	1 × 1/5	20	0 × 1/5	0
5	7	Dead	Censored	0 × 1/3	25	Competing event	0 × 1/5	20	1 × 1/5	40
4	10	Alive	Censored	0 × 1/2	25	Censored	0 × 1/5	20	0 × 1/5	40
1	10	Alive	Censored	0 × 1/1	25	Censored	0 × 1/5	20	0 × 1/5	40

A Patient B Follow-up time (years) C Status at the end of follow-up D Status E Contribution F Cumulative implant failure (%) G Status H Contribution I Cumulative implant failure (%) J Contribution K Cumulative death %

In contrast to the cumulative incidence method, the KM method excludes censored patients from the at-risk population at the time of censoring. It is assumed that these censored patients have the same probability of revision as patients who are still under observation. The assumption may, of course, be true for patients who are alive and unrevised at the end of follow-up, but is is not true for patients who have been censored because of death.

With the data in the example, the KM method estimates the cumulative revision risk to be 25% at 10 years. The cumulative incidence approach estimates the revision risk to be 20% at 10 years.

The situation becomes more complicated if there is more than one competing risk event—for example, if patients undergo revision for other causes. Cumulative incidence is the appropriate method for estimation of the survival of the implant as an independent event. However, in clinical situations—such as when different severe comorbidities are present—the patient may need a revision, but it is contraindicated. The comorbidities should then, in principle, also be considered a competing risk because they alter the probability of the revision of interest. However, if data on comorbidity are not available, then cumulative incidence estimates based only on death, with other revisions as competing risks, may not be unproblematic.

In the presence of competing risk events, cumulative incidence curves for groups can also be compared using a special log-rank test for equality of cumulative incidence curves across groups, which was developed by Gray (1988).

### Example 2. The Danish Hip Arthroplasty Register

**Figure 1.**
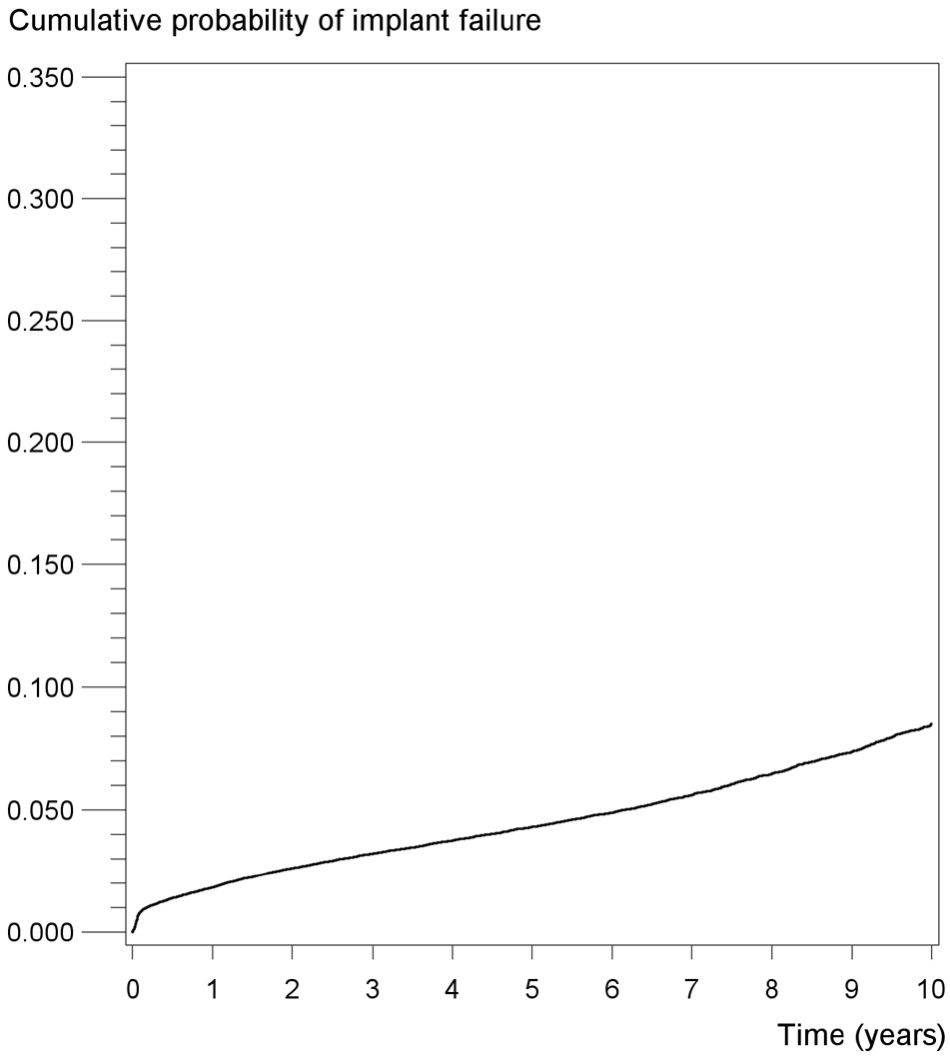
The probability of implant failure after primary total hip arthroplasty plotted against time using Kaplan-Meier estimate.

**Figure 2.**
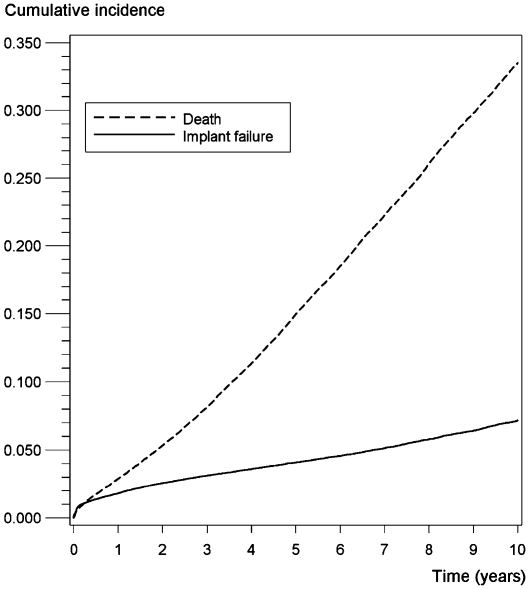
The probability of implant failure after primary total hip arthroplasty plotted against time using cumulative incidence estimate. Death is considered as the / a [authors: please choose one alternative] competing event.

Between 1995 and 2008, the Danish Hip Arthroplasty Register collected a dataset of 84,843 hip replacement procedures. At the 5-year follow-up, 11.4% of the patients were dead. 5 years later, the corresponding proportion had increased to 18.4%. At 5-year and 10-year follow-up, the KM estimate of implant failure was 4.3% and 8.5%, whereas the cumulative incidence estimates were 4.1% and 7.2%, respectively (Figures 1 and 2).

This illustrates the impact of length of follow-up on KM estimates: as the proportion of competing risk events is large and increases with follow-up time, the KM estimates become more biased. The following question arises: “Is the difference between 4.1% and 4.3% at 5 years or between 7.2% and 8.5% at 10 years clinically important?”.

### Different standpoints

These two examples show that cumulative incidence is an adequate measure to use for estimating the survival of implants without incorporating bias from patient survival.

However, from the patient's point of view, when he or she is contemplating whether or not to undergo joint replacement surgery, it is adequate to consider only what happens during the lifetime of the patient. From this standpoint, the KM method is an adequate method to use because it is based on an implicit assumption that the patient will be alive until the implant fails. The KM estimate thus gives a more logical, understandable, and clinically relevant survival estimate.

Even so, in order to make a decision on allocation of economic resources to joint replacement surgery or organization of the healthcare system, the cumulative incidence method may be more appropriate because patients who have died do not count in the risk of implant failure and will not need surgery.

### Interpretational problems

The interpretation of hazard rate ratios from Cox regression analysis is not straightforward with competing risk events, because those patients who fail from competing risk are removed from the population at risk of the event of interest.

One alternative is to compare the cumulative incidence functions using the Fine and Gray proportional hazards model. This is based on competing risk regression of the cumulative incidence functions (Fine and Gray 1999).

The method is implemented in S-PLUS, R, and Stata 11. More recently, Klein and Andersen (2005) have introduced a new regression model of competing risk data, which is available as a SAS macro. The R package for multi-state models can also be used to analyze competing risk problems; see Putter et al. (2007).

### Recommendation

When there are competing risks, survival estimates from the KM method are biased, and implant survival is overestimated. However, in data from arthroplasty registers, the size of the bias may not be clinically significant (Gillam et al. 2010).

Furthermore, the decision of whether to use the KM method or the cumulative incidence method depends on the research question to be answered. The KM estimates of implant failure are more clinically meaningful and straightforward to interpret for clinicians and patients.

The competing risk problem should be acknowledged when competing risks exist; and if KM survival estimates are presented instead of cumulative incidence estimates, the number and type of censored observations should at least be described.

## 2. The proportional hazards (PH) assumption

The Cox regression model relies on hazards being proportional; see Part I. This means that the hazard ratio (relative risk), e.g. calculated when comparing two hip implants, is constant over time. The estimated hazard ratio is biased when this assumption is violated, for example, when the survival curves cross. Alternative analyses will then be required.

### Causes of non-PH

One reason for non-PH is that a factor's effect on survival changes over time. This is, for example, often the case with implants that differ in design or materials. While they may perform similarly for the first few years after insertion, differences may become evident with prolonged follow-up, which is known to happen with some cup implants where problems associated with wear and loosening do not result in increased revision rates until at least 5–10 years after insertion (Havelin et al. 1995 a, b, 2002). Until wear and prosthetic loosening becomes symptomatic, cups that are inherently prone to these problems might still be doing just as well as cups with better long-term results (Hallan et al. 2010).

Another reason for non-PH may be incorrect modeling—for example, if important risk factors are omitted from a regression model, or the functional form of a risk factor is incorrectly specified. One example of this is the inclusion of age as a continuous variable in a log-linear model of the effect of age on revision rate. If the actual relationship is J-shaped instead of log-linear, the model is mis-specified and one consequence may be non-PH. A better alternative might then be to use indicator variables to specify what age groups patients belong to. It is also possible to model non-linearity using spline functions (Therneau and Grambsch 2000).

### The consequence of non-PH

If the PH assumption is violated, by hazard ratios increasing over time, the overall hazard ratio for the risk factor will be overestimated. Decreasing hazard ratios will lead to underestimation (Schemper 1992).

The statistical power of the corresponding tests of risk factors has also been shown to be reduced with non-PH (Lagakos and Schoenfeld 1984).

### How departures from the non-PH assumption can be investigated

For categorical variables, a simple informative graphical display can be made to evaluate the PH assumption, a log-minus-log plot. The plot shows survival curves for each value of the investigated variable plotted, on a log-minus-log scale, during follow-up. A log transformation of the time axis is often used. If the PH assumption is fulfilled, the plotted curves are approximately parallel and the vertical difference is equal to the log hazard ratio, i.e. the regression coefficient.

**Figure 3.**
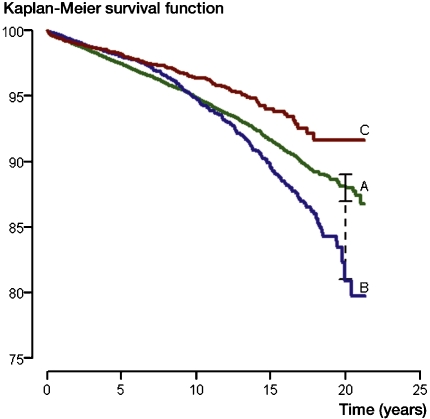
Kaplan-Meier survival curves for implants A, B, and C with standard (solid line) and modified (dotted line) 95% confidence limits for implant A at 20 years. follow up.

**Figure 4.**
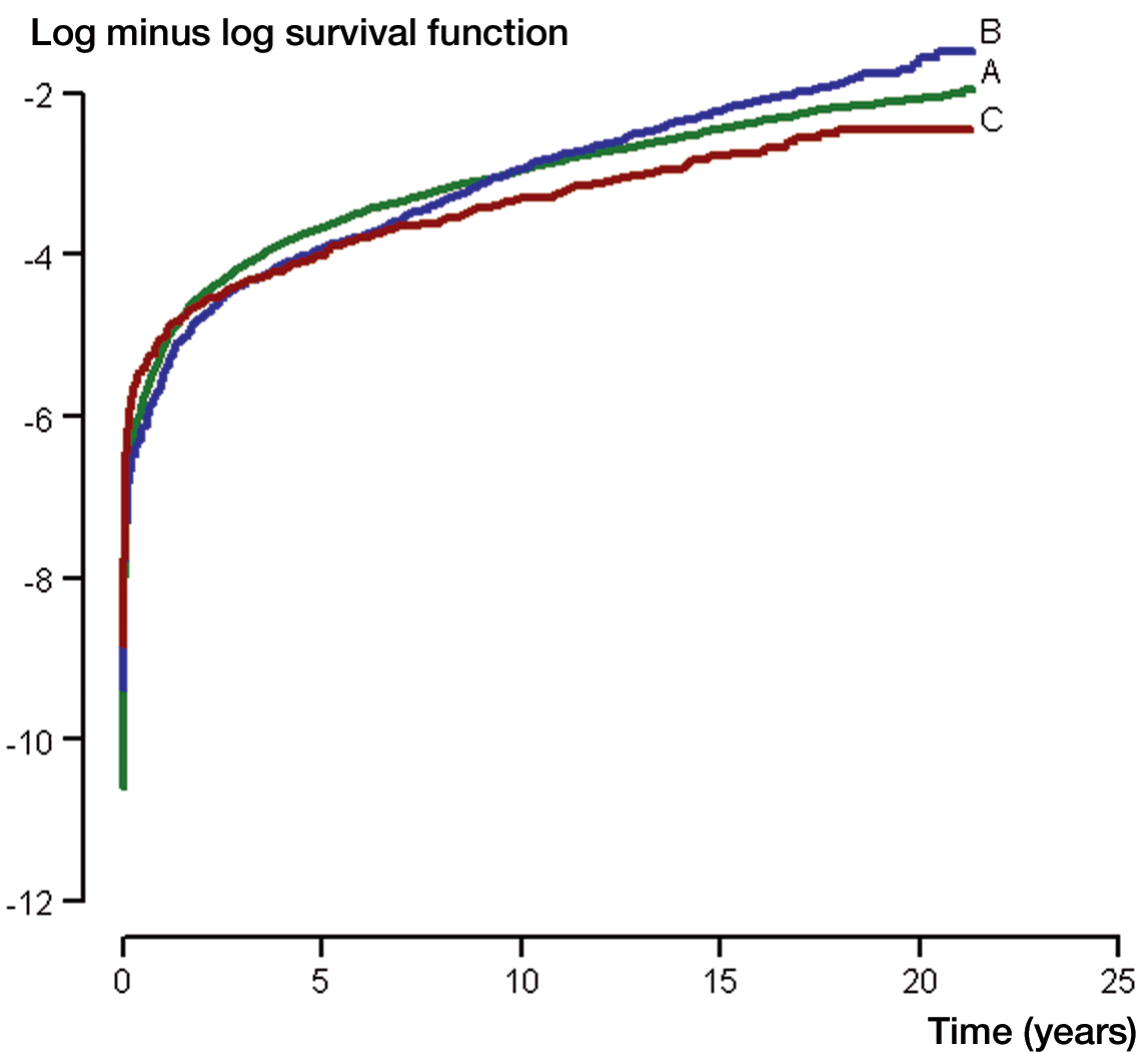
Log-minus-log Kaplan-Meier survival curves for implants A, B, and C.

Consider, for example, 3 hip implants: A, B, and C. Their Kaplan-Meier survival curves (Figure 3) indicate that the PH assumption does not hold for a comparison of implant A and B. This is also evident from the log-minus-log plot (Figure 4), where the vertical distance separating the curves clearly varies with time since operation.

However, it is often difficult to decide whether the variation is large enough to conclude whether hazards are proportional or not. The relation between implant C and implant A is an example of this (Figure 4). The number of implants in some of the groups may also be small, and contribute to the uncertainty. Furthermore, the curves plotted will be based on few observations towards the ends of the curves.

Another graphical approach is based on the notion of time-varying regression coefficients *ß(t)*. If the PH assumption is fulfilled, *ß(t)* is equal to a constant c throughout follow-up, and a plot of *ß(t)* against time will therefore be a horizontal line that cuts the y-axis at c. Such a plot may be based on scaled Schoenfeld residuals; this is also known as a partial residual plot (Grambsch et al. 1995).

**Figure 5.**
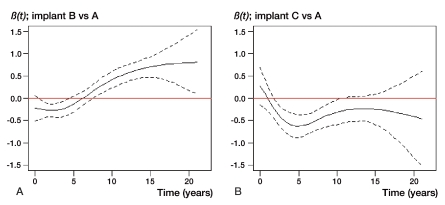
Smoothed scaled Schoenfeld residuals (solid line) with 95% confidence limits (dotted lines) are given for comparison of implant B with implant A (panel A) and for comparison of implant C with implant A (panel B). The graphs show that while early survival of implant B is better than that of implant A, survival of implant B is inferior with longer follow-up, and that survival of implant C is consistently better than that of implant A. The horizontal red line indicates no difference in hazard rates (*ß(t)* = 0 for all values of t or equivalent that the relative risk is equal to 1).

Test statistics have been developed to test the null hypothesis of a constant regression coefficient over time (Therneau and Grambsch 2000). The scaled Schoenfeld residual plot for implant B versus implant A indicates that while the early survival of implant B is better than that of A, B is inferior with longer follow-up (Figure 5a). The null hypothesis of *ß(t)* being constant over time is also rejected (p < 0.001) (; row “overall”).

Figure 5b shows *ß(t)* estimated for implant C versus A. Although C appears to be inferior to A shortly after operation, the difference is not statistically significant—as evaluated using the 95% confidence limits. Instead, the overall impression is that implant C is consistently better than A. This is also confirmed by the non-PH test result giving a p-value equal to 0.5 (Table 2; row “overall”).

**Table 2.** Relative risk (RR) estimates based on Cox regression analyses for all observations (overall) and with restriction on survival times with cutpoints set at 5 and 10 years

Implant:	A	B				C			
Follow-up	RR	RR	95% CI	p-value	non-PH	RR	95% CI	p-value	non-PH
Overall	1	1.08	0.98–1.19	0.1	< 0.001	0.72	0.62–0.83	< 0.001	0.5
0–5 years	1	0.77	0.66–0.90	0.001	0.9	0.73	0.60–0.89	0.002	0.003
5–10 years	1	1.19	1.00–1.42	0.06	0.001	0.71	0.54–0.92	0.01	0.6
> 10 years	1	1.67	1.39–1.99	< 0.001	0.1	0.70	0.50–0.97	0.03	0.5

### Dealing with non-PH

Several approaches to dealing with departures from the non-PH assumption are available and can be performed within the context of the Cox model.

#### Stratified analyses

A risk factor that is unrelated to the PH assumption may be included in the model as a stratification factor. The main drawback of this is that it will no longer be possible to estimate the relationship between this risk factor and survival. In the example, implant brand is the main risk factor and stratification is therefore not an option. It is also a disadvantage that stratified analyses are less efficient than unstratified (Therneau and Grambsch 2000).

#### Time axis division

If the PH assumption is fulfilled for specific follow-up intervals, the Cox regression model can be fitted with restricted follow-up. For instance, if it is indicated that there are no departures from the PH assumption during the first 5 years of follow-up, follow-up could be restricted to this interval, by censoring implants still at risk beyond 5 years.

The time axis may also be partitioned into several time intervals (Havelin et al. 2002, Espehaug et al. 2009). For the example comparing implants A, B, and C, overall results and results within time intervals are given in Table 2.

When implant B is compared with implant A, the reference implant, the overall hazard ratio (relative risk) estimate does not indicate any difference in survival between the two implants. However, the time-dependent analyses show that while the short-term result of implant B is better than that of A, the long-term results of B are worse than those of A.

A test of non-PH within each time interval indicates proportionality for the first and last interval. The results of comparison of implant C and implant A are also in accordance with what was stated earlier.

While such analyses can be informative, the results clearly depend on the choice of time intervals. The statistical power for each analysis will also be reduced as fewer event times are included in each analysis. Thus, the procedure should not be recommended for small sample sizes or for samples with heavy censoring (Schemper 1992).

#### Time-dependent coefficients

It is possible to model time-varying effects by creating time-dependent risk factors (*X(t)*), which can be included in a Cox model. Many functional forms of *X(t)* can be chosen. It may be difficult to decide on the form of *X(t)*, but the decision can be based on theoretical clinical assumptions, or be motivated by findings from the analysis performed in the investigation of PH. However, this implies searching for statistically significant findings, and the exploratory nature of such results should be recognized. A division of the time axis may also be easily modeled as time-dependent risk factors using heaviside (or unit step) functions (Kleinbaum and Klein 2005).

#### Schemper's weighted model

An alternative to the previously described methods is Schemper's model, which in contrast to Cox's model weights observations differently depending on follow-up (Schemper et al. 2009). While the Cox model gives the same weight to early and late hazard ratios, which biases the hazard ratio, the weighting in Schemper's model enables the estimation of a hazard ratio representative of overall follow-up.

#### Other approaches

All of the methods described here represent extensions of the Cox regression model. There are many other models for survival data that can be used, however, including different parametric models, the accelerated failure time model, and the additive hazards model (Aalen 1989, 1993).

### Recommendation

It is necessary to investigate the fulfillment of—and to correct for departures from—the assumption of PH. Standard statistical program packages usually offer several solutions to the problem. A simple initial analysis would be to create log-log plots, but hypothesis tests of Schoenfeld residuals are less subjective.

The methods described for detection of non-PH may be of more use as tools, however, in an exploratory investigation of the form of time-varying coefficients than as a means of testing a simple hypothesis of PH. In many studies, non-PH represents an important finding in itself, which should be explored further. Several papers based on registry data have demonstrated this (Johnsen et al. 2006, Gjertsen et al. 2007, Espehaug et al. 2009, Schrama et al. 2010).

In registry studies based on relatively large samples, the analyses can be performed most easily with a partition of the time axis, or by including time-dependent variables in the Cox regression model. Should a summary measure of the overall hazard ratio, adjusted for non-PH, be of primary interest, the weighted model of Schempers et al. (2009) may be a better alternative.

## 3. Bilateral observations

Bryant et al. (2006) reported that a high proportion (42%) of clinical studies in orthopedical journals generally involve inappropriate use of multiple observations from single individuals.

Patient-specific physiological and behavioral factors can be expected to play an important role in the lifetime of prosthesis (Robertsson and Ranstam 2003). For example, patients with bilateral coxarthrosis tend to strain the painless hip, which can cause loosening of the prosthesis (Möllenhoff et al. 1994).

Many statistical methods, including Kaplan-Meier analysis and Cox's model, are based on an assumption of independent observations. However, multiple observations on one patient, such as knees, hips, ankles, etc., usually have more in common than single observations from different patients, i.e. within-subject measurements often have lower variance than between-subject measurements. This implies that such observations are not independent but correlated. The correlation is known as intraclass correlation.

The consequence of violating the assumption of independent observations is often that the statistical precision is overestimated, with p-values being too low and confidence intervals too narrow (Ranstam 2002).

Several methods are available for handling the problem. The simplest one is to include only one observation from each patient. However, an alternative is to analyze the correlated observations using a method that allows inclusion of correlated observations, e.g. by including a shared frailty variable in the Cox regression model (Hougaard, 2000, Robertsson and Ranstam 2003), by fitting a marginal model (Carriere and Bouyer 2002), or by using resampling techniques (Hoffman et al. 2001).

For practical reasons, the discussion here will be restricted to two alternatives: (1) including only one observation per patient, and (2) fitting a shared gamma frailty model (Cox model with a shared frailty variable assumed to have a gamma distribution).

The basic assumption of frailty models is that the dependency in the failure times of correlated observations (of a bilaterally treated patient) can be ascribed to an unobservable, latent, patient-specific variable, and that the failure times of a patient are independent when the analysis is conditioned on this (Schwarzer et al. 2001).

Results of analyses of data from arthroplasty registers usually disregard bilaterality problems. It is simply assumed that the revision risks of uni- and bilateral prostheses are identical (Havelin et al. 2000, Malchau et al. 2002, Eskelinen et al. 2005). In spite of this, there has not been any generally accepted view on the effects of ignoring bilaterality (Robertsson and Ranstam 2003).

### Review of the literature

Visuri et al. (2002) studied the influence of bilaterality on the survival of hip prostheses using data from the Finnish Endoprosthesis Register. The material was divided into 4 study groups: unilateral THA, first and second bilateral THA, and 1-staged bilateral THA. The survival of the first bilateral prosthesis was similar to that of unilateral prostheses. However, the second bilateral prosthesis survived statistically significantly longer than unilateral prostheses. The authors concluded that the better survival of the second bilateral results in too favorable a prognosis, and that the bias could be expected to increase with the proportion of bilateral prostheses.

Havelin et al. (1995) studied patients with Charnley prostheses based on data from the Norwegian Arthroplasty Register. Separate analyses were performed to examine the effect of bilateral replacement as opposed to unilateral replacement on prosthesis survival. The overall results for patients who had had a unilateral operation were not statistically significantly different from the overall results for those who had had a bilateral operation. It was concluded that the possible effect of dependencies within a patient was unimportant.

In their study of estimation of frailty models, Ripatti and Palmgren (2000) used data from male patients who had undergone primary total hip arthroplasties. Over 20% of the patients received bilateral implants and 30% of the patients had more than one operation. Two different frailty structures were fitted: a shared frailty model and a hierarchical frailty model. The more flexible frailty model gave estimates closer to those of the Cox model, ignoring the dependencies in the data. It was concluded that a tight model structure for the dependence in the shared frailty model might induce model misspecification bias in the fixed-effects estimates.

The bias of not taking bilateral operations into account was also investigated by Robertsson and Ranstam (2003), by analyzing patients who underwent knee arthroplasty surgery in Sweden, using both a traditional proportional hazards analysis and a shared gamma frailty model. Comparison of revision risk between TKA and UKA and ignoring bilaterality by using a traditional proportional hazards analysis yielded a hazard ratio estimate of 1.84 (95% CI: 1.71–1.97). Accounting for subject dependency among bilateral prostheses, by performing the comparison using a shared gamma frailty model, yielded a hazard ratio estimate of 1.98 (95% CI: 1.83–2.14). The authors' conclusion was that the effect of ignoring subject dependency of bilateral operations is negligible.

Schwarzer et al. (2001) also used a shared gamma frailty model to model bilateral dependencies for primary hip prosthesis data, and they concluded that failure times of bilateral hip prostheses could be treated as if they were independent when relevant prognostic factors were considered in the analysis.

In the study by Lie et al. (2004), information from the Norwegian Arthroplasty Register was used. The results from an ordinary Cox analysis were compared with the results from a marginal model, a shared gamma frailty model, and a model using a time-dependent covariate to condition from failures in the opposite hip. No practical difference between the three calculated survival curves for the hip replacement data was found. It was concluded that in analyses of prostheses survival, the dependencies between bilateral observations should be considered, but ignoring the dependency does not necessarily have any effect on the results.

### Recommendation

In several studies, the dependency of bilateral prostheses has been shown to have little practical consequences, at least with hip and knee data. Inclusion of only one side of the bilaterally operated patients solves the dependency problem, but it can also induce bias and does lead to loss of statistical power. Notwithstanding these remarks for hip and knee data, it is generally important to be aware of possible problems with dependent data. Consequences may be more serious for other joints. The number of independent observations and bilateral observations should always be presented. Sensitivity analyses may be useful to show that results are robust regarding departures from the independence assumption.

## 4. Revision rate ranking

Nordic arthroplasty registers generally have data of high quality and excellent coverage. Arthroplasty registers usually also provide information on revision rates for different types of implants and for patients with different diagnoses. It may therefore seem straightforward to use ranking as a means of identifying clinically important differences between clinics and at the same time establish benchmarks for optimal treatment.

Ranks of observed revision rates are not easily interpreted, however. First, in contrast to the revision rates of clinics, ranks are seldom presented with any information on sampling uncertainty, e.g. with confidence intervals. Secondly, the consequences of less than perfect data quality, i.e. random and systematic misclassification of revised patients as unrevised, are usually not considered. Thirdly, the sample of patients being treated at different clinics is usually unbalanced with respect to predictive factors, which—with the same quality of care—would also cause differences in outcome. This problem is often referred to as a case mix problem.

Given the generally low revision rates in Nordic knee and hip arthroplasty registers, which in itself makes it difficult to identify clinically important differences between clinics, each one of the three sources of spurious rank differences has the potential to confuse ranks substantially. Results from comparisons based on ranking of clinics thus carry less useful information than would be expected, and are prone to misunderstandings.

A more detailed description of the three different rank confusion phenomena, with suggestions on how to assess and reduce the uncertainty in rank comparisons, is presented below.

### Sampling uncertainty

Sound generalization of scientific findings, to subjects other than those examined, accounts for the fact that the representativeness of observations in a sample is uncertain. Traditionally, the influence of this uncertainty on the findings made is evaluated with hypothesis tests and presented to the reader in terms of p-values.

For many years, however, it has been argued that interval estimation with presentation of uncertainty using confidence intervals is a better alternative. The Uniform Requirements for Manuscripts Submitted to Biomedical Journals (ICMJE) also state that findings should be quantified and presented with appropriate indicators of uncertainty, and confidence intervals are suggested.

The influence of sampling uncertainty on a hopital's rank number cannot usually be calculated using conventional statistical methods. Confidence intervals for the ranked observations may be presented, however. These confidence intervals unfortunately do not describe rank uncertainty directly, and should not be used for this purpose.

Confidence intervals for ranks can, however, be calculated using Monte Carlo simulation (Marchall and Spiegelhalter 1998), which is easily achieved with modern computers. For ranking of hospitals with regard to revision rates, the confidence interval calculations would be performed on the basis of hospital-specific revision rates and their sampling uncertainty.

Revision rates of knee and hip prostheses are generally low. As a consequence, the information on hospital-specific revision rates may be very limited for hospitals performing few primary operations. This may, in turn, lead to exaggeration of differences in revision rates between hospitals. Assume, for example, that a hospital performs just 1 primary operation. Whatever the true revision rate for the hospital, the observed revision rate can only be 0% or 100%, depending on whether the prosthesis is revised or not. Of course, such randomly exaggerated hospital effects have great impact on hospital ranks.

Two basically different methods are available for estimation of hospital-specific revision rates. These treat hospital-specific revision rates either as fixed or random effects. While the former corresponds to estimating revision rates using a Cox model, the latter corresponds to using a shared frailty model. Analysis of revision rates as random effects has the advantage of protecting against exaggerated differences as described above (Robertsson et al. 2006).

### Misclassification

As much as all registries have routines for monitoring registration procedures and validating data, no registry has error-free data only. For continuous data, the term accuracy is used to describe the correctness of the information registered. For categorical data, registration errors that lead to an erroneous category being registered are known as misclassifications. For example, when a prosthesis revision is erroneously registered as a primary operation, there has been a misclassification.

Many prosthesis registries do not register revisions directly, but define revisions by deriving information from comparisons of two or more operation records on the same patient; if a subject has a second operation on the same side, then the second operation is considered a revision.

Clerical errors in registration of patient ID number, side, or date of operation may then result in a misclassification of a revision as a primary operation. The revision rates observed are thus uncertain—not only because of sampling, but also because of measurement errors.

Valid ranking of hospitals requires valid data. Monte Carlo simulation of misclassification in the Swedish Hip Prosthesis Registry (Ranstam et al. 2008) showed that misclassification probabilities as low as 2–3%, although they had minor consequences for revision rates, had major effects on hospital ranks. Hospital-specific revision rates are very similar.

It is therefore doubtful whether registries can achieve data that are of high enough quality for valid ranking. Investigation and presentation of the validity of registry data should be prioritized in registries used for hospital ranking.

While sampling uncertainty, at least under the assumption of random sampling, is usually considered an entirely random phenomenon, misclassification can also be assumed to have a systematic component. This could, for example, be the case if revision registration from a hospital with high revision rates was prone to a large number of registration errors, leading to fewer revisions being identified—thus giving the hospital a better rank than it deserves.

### Case mix

The term case mix refers to the mix of cases treated by a hospital. As the allocation of cases to hospitals is not randomized, it is plausible, or even likely, that cases treated by different hospitals will have systematic differences in predictive factors such as age, sex, severity, and so on. One could, for example, hypothesize that severe cases have a greater propensity to be treated at university hospitals than less severe cases.

The effect of an unbalanced case mix is that estimated revision rates are confounded by association with the predictive factors. This bias will, of course, also have consequences for the validity of the hospital ranks, giving some hospitals erroneously low—and others erroneously high—ranks.

It is possible to adjust revision rates for the unbalanced case mix using statistical models, if all predictive factors are known and registered. This is seldom the case, however. It is more probable that not all the predictive factors are known, and only a few of all the known predictive factors are usually measured and registered.

Even when statistical case mix adjustments have been performed, it is reasonable to expect that confounding effects remain.

### Clinical relevance

It is of value to document different outcome parameters over time. It is especially beneficial for the individual hospitals or operating units to analyze their results on a continuous basis. If the aim is to ascertain, maintain, and even improve the quality of a medical intervention, the results have to be known. It is also important to know the expected results based on a large sample average, if such information is available. If, for example, the overall revision rate within 2 years of a knee arthroplasty doubles, this could either be true or spurious variation. Even if the true nature of this increase cannot be established, further studies of these patients may be helpful to improve future practice. Perhaps one or several steps in the treatment of these patients may be inadequate or fails to work as intended. Experiences from the Swedish total hip arthroplasty register point in that direction.

The information as such will raise a number of questions. The first question is whether this increase is really true or just a result of random variation? Has the patient population or case mix changed? As indicated above, it is difficult to do a proper statistical evaluation of this problem. For high-volume hospitals, and especially those with a comparatively uniform patient population, an answer based on sound statistics may be possible. For units with low volumes and infrequent outcomes, the true nature of variations in outcome on an annual basis can rarely be answered.

Regardless of whether or not there is statistical significance, increasing incidence of an undesirable outcome should alert the health providers to perform an in-depth analysis. This evaluation could be very simple and should at least include a case-by-case analysis. Such an analysis will provide valuable information, especially if inferior results turn out to be caused by avoidable clinical mistakes and malpractice.

The outcome of, for example, a hip arthroplasty will affect the patient in a multitude of ways, and for a proper analysis several outcomes must be measured. These outcomes should be valid, should be easy to define and to measure, and should include the opinion of the patient. Some of them, e.g. patient-reported outcome parameters such as EQ-5D, might be easier to treat from a statistical point of view, but are still dependent on sample size and case mix.

In several countries, it is mandatory to report outcomes of medical interventions at the hospital level. The idea is to encourage and stimulate continuous improvement. There is also a demand from the public to gain access to this information. This development will increase the demand on the profession to provide simple instruments for interpretation of such data. As already mentioned, a proper and meaningful statistical evaluation that ranks performance at the hospital level is difficult and sometimes impossible due to small sample size, lack of sufficient information, and missing or incorrect information. On the other hand, continuous monitoring of results from individual hospitals can be extremely valuable and is a prerequisite for persistently high quality of healthcare. In that process, comparisons with expected performance become more or less unavoidable. It is, however, important that the uncertainty of such comparisons is recognized and that the accuracy of the comparisons is assessed to minimize the risk of misinterpretation.

### Recommendation

Ranking of hospital-specific outcomes such as revision rates is associated with several methodological problems and should be considered to be an uncertain method for comparison of hospitals. If done, the inherent uncertainties should be described as clearly as possible: rank numbers should be presented with confidence intervals, data validity or misclassification probabilities should be investigated and described, and the influence of confounding factors should be discussed and accounted for as far as possible.
